# A Case of Painless Subacute Thyroiditis Presenting as Fever of Unknown Origin

**DOI:** 10.7759/cureus.24949

**Published:** 2022-05-12

**Authors:** Binita Neupane, Sunita Karki, Ekta Tirthani, Nagesh Jadhav, Nisha Gupta

**Affiliations:** 1 Internal Medicine, Rochester Regional Health, Rochester, USA; 2 Internal Medicine, BronxCare Health System, New York City, USA

**Keywords:** painless, subacute thyroiditis, self limited, fever of uknown origin, painless thyroiditis

## Abstract

Subacute thyroiditis (SAT) is characterized by severe pain in the anterior aspect of the neck and tenderness is present during the thyroid gland's palpation. It is commonly caused by viruses including mumps, measles, rubella, coxsackievirus, influenza, and Epstein-Barr virus (EBV). Painless subacute thyroiditis is rare and can present as a fever of unknown origin (FUO). Our case reports an unusual case of SAT as our patient did not have any neck pain. Laboratory investigations show low thyroid-stimulating hormone (TSH), poor or no uptake of radioactive iodine by the thyroid, and elevated erythrocyte sedimentation rate (ESR). Clinicians should be aware that painless SAT can present as a fever of unknown origin.

## Introduction

Subacute thyroiditis (SAT) is characterized by severe pain in the neck's anterior aspect and is commonly caused by viruses. Hyperthyroidism symptoms like tremors, heat intolerance, anxiety, weight loss, and increased frequency of bowel movements can be present. Painless SAT can occur rarely and present as a fever of unknown origin (FUO). 

## Case presentation

A 40-year-old male, a physician by profession, called his primary care doctor's office with the chief complaints of fever of a few days duration associated with body aches, dry cough, back pain, and drenching night sweats. He was prescribed oseltamivir over the phone. However, the fever continued, making him go to the urgent care where a chest x-ray was done and was normal. On day eleven of illness, the fever persisted, which prompted him to go to the emergency department; where he was observed to be tachycardic and intravenous fluids were administered. He was noted to have a mild elevation of aspartate aminotransferase (AST) and alanine transaminase (ALT). Past medical history was significant for positive purified protein derivative (PPD) since 2006, stable liver hemangioma in MRI abdomen since 2017, and childhood asthma. Four weeks before the illness, he had an upper respiratory tract infection, and his wife and child had such symptoms one week before his illness. No recent travel history. His family history was notable for hypothyroidism in his sister. On physical examination, vitals were significant for a fever of 100.3 F, and a pulse rate of 107 beats per minute. There was no tenderness and nodules on thyroid gland palpation. Bruits were not appreciated. Otherwise, the physical examination was normal.

Blood work was notable for white blood cell count: 5900/µL (normal: 4.0-10 x 10^3^/µL) with a normal differential count. AST:44 (normal: 0-35 units/L), ALT: 94 (normal: 0-35 units/L), C reactive protein (CRP) 52.5 (normal: 0.0-8.0 mg/L), and ESR: 83 (normal: male 0-15 mm/h) (Table 1). Urine analysis was clear. Chest x-ray was normal. A liver ultrasound revealed a stable size of liver hemangioma measuring 2.1 cm. An infectious disease specialist was consulted as the fever continued, and an infectious workup as follows was ordered. Monospot test, HIV 1 and 2, and blood cultures were negative. Cytomegalovirus IgG antibody was positive, and IgM antibody was 10 (normal:<29.9 AU/ml). The atypical viral panel was negative. However, the fever and tachycardia continued to persist; a thyroid function test (TFT) was then added to the admission labs, which showed high free T4 at 2.9 (normal: 0.9-2.4 ng/dL), T3 at 182 (normal: 70-195 ng/dL), and low TSH at 0.07 (normal: 0.5-5.0 µU/mL). Anti-thyroid peroxidase (anti-TPO) antibody was normal at less than 28 (normal: <60 IU/ml). Anti-thyroglobulin antibody was within the normal range at 54.2 (normal: <60 IU/ml). A radioiodine thyroid uptake scan revealed low uptake consistent with subacute thyroiditis. He was treated with non-steroidal anti-inflammatory drugs (NSAIDs) for three days without much relief so he was started on prednisone. Fever, fatigue, and drenching night sweats subsided after prednisone was initiated. TFTs were checked six weeks after his initial presentation normalized. 

**Table 1:** Laboratory values on the day of evaluation and six weeks after the treatment

Labs	Values on the day of evaluation	Values six weeks after treatment
White blood cell count (WBC)	5900 (normal: 4000-10,000 µL)	
Aspartate aminotransferase (AST)	44 (normal: 0-35 units/L)	
Alanine transaminase (ALT)	94 (normal: 0-35 units/L)	
C-reactive protein (CRP)	52.5 (normal : 0.0-8.0 mg/L)	
Erythrocyte sedimentation rate (ESR)	83 (normal: male 0-15 mm/h).	
Free thyroxine (free T4 )	2.9 (normal: 0.9-2.4 ng/dL)	1.4 (normal: 0.9-2.4 ng/dL)
Triidothyronine (T3)	182 (normal: 70-195 ng/dL)	112.9 (normal: 70-195 ng/dL)
Thyroid-stimulating hormone (TSH)	0.07 (normal: 0.5-5.0 µU/mL)	1.88 (normal: 0.5-5.0 µU/mL)

## Discussion

FUO, first reported in 1961 by Petersdorf and Beeson, is one of the diagnostic challenges of internal medicine [1]. FUO is characterized by a temperature of 101 degrees Fahrenheit (38.3 degrees Celsius) or higher for at least three weeks without a proven diagnosis despite a minimum of one week of workup in the hospital [2]. Endocrine disorders are a very rare cause of fever of unknown origin [3]. About 5% of thyroid disorders are comprised of subacute thyroiditis [4]. 

SAT is a self-limited inflammatory disorder of the thyroid gland, characterized by sudden neck pain and thyrotoxicosis. Symptoms include viral prodromes like myalgia, malaise, and fatigue [5]. Viruses are thought to induce subacute thyroiditis. Viruses commonly associated are mumps, measles, rubella, coxsackievirus, influenza, and EBV. There is often no leukocytosis, and after a few weeks or months, complete recovery occurs. SAT is associated with severe pain in the neck's anterior aspect, and tenderness is present on the thyroid gland's palpation. Laboratory investigations show low TSH, poor or no uptake of radioactive iodine by the thyroid, and elevated ESR [6]. In a cohort study done in Minnesota among 160 patients, 94% had pain as a presenting symptom for SAT [7].

There are three stages of SAT: The first stage can manifest as hyperthyroidism, due to the destruction of thyroid tissue. The second stage presents as transient hypothyroidism, affecting around 30% of patients. The third stage is euthyroidism, which typically lasts 12 months. Of the patients, 5-26% might develop permanent hypothyroidism requiring lifelong thyroid hormone replacement [8]. Management options vary; some people do not require treatment, while others require NSAIDs. Corticosteroids are needed to treat individuals who do not improve on NSAIDs and those with severe symptoms. Corticosteroids usually are provided only after acute suppurative thyroiditis has been ruled out [6]. In a study of 150 patients in Nepal with SAT, treatment with oral prednisolone at a dose of 20 mg, tapered over four weeks, resulted in pain alleviation in 94% of the patients in just two weeks. It also resulted in a 96% drop in ESR to normal levels after four weeks [9].

Thyroid autoantibodies have a low correlation with disease course and can be present in a minority of patients. The transient immunological response can occur as a secondary phenomenon for SAT, but autoimmunity is not a primary factor for SAT initiation [10].

## Conclusions

FUO is commonly encountered as a diagnostic challenge. Endocrine disorders are a rare cause of FUO; with SAT comprising 5% of thyroid disorders, the chance of it being overlooked is possible. Common symptoms of SAT are usually viral prodromes like myalgia, malaise, fatigue, and neck pain. Of the patients with SAT, 94% present with a painful neck. However, we should be mindful that painless SAT is a possibility. Our case was unusual as our patient did not have any neck pain. Clinicians should be aware that painless SAT can present as FUO.
